# Identification of genetic variants or genes that are associated with Homoharringtonine (HHT) response through a genome-wide association study in human lymphoblastoid cell lines (LCLs)

**DOI:** 10.3389/fgene.2014.00465

**Published:** 2015-01-13

**Authors:** Yin Tong, Nifang Niu, Gregory Jenkins, Anthony Batzler, Liang Li, Krishna R. Kalari, Liewei Wang

**Affiliations:** ^1^Department of Hematology, Shanghai General Hospital, Shanghai Jiao Tong University Affiliated First People's HospitalShanghai, China; ^2^Department of Molecular Pharmacology and Experimental Therapeutics, Mayo ClinicRochester, MN, USA; ^3^Division of Statistic and Bioinformatics, Department of Health Science Research, Mayo ClinicRochester, MN, USA; ^4^Department of Oncology, Institute of Medicinal Biotechnology, Peking Union Medical College and Chinese Academy of Medical SciencesBeijing, China

**Keywords:** genome-wide association study, biomarkers, Homoharringtonine (HHT), lymphoblastoid cell line system, leukemia

## Abstract

Homoharringtonine (HHT) has been widely used in China to treat patients with acute and chronic myeloid leukemia for decades. Since response to HHT varies among patients, our study aimed to identify biomarkers that might influence the response to HHT using a panel of various human lymphoblastoid cell lines (LCLs). Genome-wide association (GWA) analysis using single nucleotide polymorphism (SNP) and mRNA expression data was assessed for association with cytotoxicity to HHT in LCLs. Integrated analysis among SNPs, expression, AUC value was also performed to help select candidate genes for further functional characterization. Functional validation of candidate genes was performed using leukemia cell lines (U937, K562). Candidate genes were knocked down using specific siRNA and its response to HHT was assessed using MTS assay. We found that 15 expression probes were associated with HHT AUC with *P* < 10^−4^, and 96 individual probe sets with *P* < 10^−3^. Eighteen SNPs were associated with HHT AUC with *P* < 10^−5^ and 281 SNPs with *P* < 10^−4^. The integrated analysis identified 4 unique SNPs that were associated with both expression and AUC. Functional validation using siRNA knockdown in leukemia cell lines showed that knocking down *CCDC88A, CTBP2, SOCS4* genes in U937 and K562 cells significantly altered HHT cytotoxicity. In summary, this study performed with LCLs can help to identify novel biomarker that might contribute to variation in response to HHT therapy.

## Introduction

Homoharringtonine (HHT) is a natural cephalotaxus alkaloid that is derived from the *Cephalotaxus* species found in China. It has been widely used in China for the treatment of acute myeloid leukemia (AML) and chronic myeloid leukemia (CML) for more than 3 decades. In the United States, the semi-synthesized HHT, known as Omacetaxine, has been approved by FDA in the setting of CML after failure of 2 or more tyrosine kinase inhibitors (TKIs) (Kantarjian et al., 2013). HHT exhibits its anti-leukemic effects in myeloid leukemia primarily through inhibition of protein synthesis, cell proliferation and by enhancing apoptosis of leukemic cells (Zhou et al., 1995; Yinjun et al., 2004; Tang et al., 2006).

Previous studies have shown that HHT has significant synergistic effects with cytarabine (Zhou et al., 1995). Based on these observations, investigators in China attempted to add HHT into the conventional induction therapy (anthracycline plus cytarabine regimen) in newly diagnosed patients with AML to improve therapeutic outcomes. A multicenter randomized controlled phase 3 trial was carried out to assess the efficacy and safety of HHT-based induction treatment. Results showed that HHT in combination with cytarabine and aclarubicin (HAA regimen) or daunorubicin (HAD regimen) improved both overall complete response (CR) rate and estimated 3-year overall survival in de novo AML patients under the age of 57 years. However, the addition of HHT also increased early death rate due to more severe marrow suppression (Jin et al., 2013). These findings raised a question of when HHT should be used in AML chemotherapy. If we can find biomarkers that can predict the response to HHT, then we can decide whether we should add HHT into conventional regimen. Selection of patients predicted to respond to HHT would maximize drug efficacy while minimizing drug-related toxicity.

Several clinical factors may influence drug response; however, the genetic polymorphism in germline genome can also play an important role in variation in the response to cancer therapy (Wang et al., 2011). In recent years, the application of high-throughput genomic techniques makes it possible to identify variation across genome that may help to find biomarkers responsible for drug sensitivity (Sachidanandam et al., 2001; Morley et al., 2004; Sabeti et al., 2007). In addition, expression quantitative trait locus (eQTL) studies performed using gene expression profiles have identified single nucleotide polymorphisms (SNPs) associated with drug response through their influence on gene expression. The integration of genotype, gene-expression data as well as drug-induced cytotoxicity or other pharmacologic phenotypes has been successfully applied in various previous studies using the same approach described in this study (Huang et al., 2007; Li et al., 2009).

This study was designed to study the role of genetic variation in variation in HHT response by using these well-established pharmacogenomic approaches that involve genome-wide basal gene expression profiles and genome-wide SNPs for 278 human lymphoblastoid cell lines (LCLs) to identify SNPs/genes that might contribute to variation in HHT drug response. This LCL model system has been successfully used to identify biomarkers for multiple chemotherapeutic agents in our laboratory (Li et al., 2008; Niu et al., 2010; Jiang et al., 2013). As described previously, we have obtained genomic information for these LCLs involving approximately 1.3 million SNPs per cell line, 54,613 mRNA expression probe sets. We also have performed genome-wide imputation using 1000 Genomes Project data in these LCLs. We next performed a HHT cytotoxicity assay with the same LCLs to obtain HHT area under the curve (AUC) as an *in vitro* HHT response phenotype. After genome-wide association (GWA) analysis involving single nucleotide polymorphisms (SNPs), mRNA data and also integrated analysis of SNPs, gene expression and AUC. The analysis resulted in 7 candidate genes that we further performed siRNA knockdown to determine their effect on HHT response or cytotoxicity. Three genes showed significant impact on HHT cytotoxicity after knocking down with specific siRNAs.

In summary, we have used genome-wide SNPs and expression data from a cell line-based model system to identified genes that were associated with HHT sensitivity. Candidate genes were then functionally validated by MTS assay. Our goals were to identify possible biomarkers that might contribute to HHT cytotoxicity.

## Materials and methods

### Cell lines

EBV-transformed LCLs from 93 African-American (AA), 91 Caucasian-American (CA), and 94 Han Chinese-American (HCA) unrelated healthy subjects (sample sets HD 100AA, HD 100 CAU, HD 100 CHI) were purchased from the Coriell Cell Repository (Camden, NJ, USA) as reported previously (Li et al., 2008, 2009; Niu et al., 2010; Jiang et al., 2013). All the samples had been anonymized by the National Institute of General Medical Sciences (NIGMS) before deposit, and all the subjects had provided written consent for their experimental use. Human leukemic monocyte cell line U937 and human erythromyeloblastoid leukemia cell line K562 were obtained from ATCC (U937, ATCC® CRL-1593.2™; K562, ATCC® CCL-243™). LCLs were cultured in RPMI 1640 medium (Mediatech, VA, USA) supplemented with 15% heat-inactivated Fetal Bovine Serum (FBS) (Atlanta Biologicals, GA, USA). K562 cells and U937 cells were grown in RPMI-1640 medium with 10% FBS.

### Drugs and cell proliferation assay

HHT was purchased from Sigma-Aldrich (St. Louis, MO, USA). HHT cytotoxicity studies were performed to determine the range of variation in HHT AUC among the 278 LCL cell lines from three ethnic groups. Drugs were dissolved in DMSO and were frozen at −20°C. Cell proliferation assay were performed in triplicate as described previously (Li et al., 2008). Briefly, 100 μl of LCL cells or U937 cells (5 × 10^6^ cells/ml) were plated into 96-well plates (Corning) and were treated with HHT concentration at 0, 2.5, 5, 10, 20, 40, 80, 160, 320 nmol/L. After incubation for 72 h, 20 μl of CellTiter 96® Aqueous MTS Cell Proliferation Assay solution (Promega Corporation, Madison, WI, USA) was added to each well. Plates were read in a Safire 2 plate reader (Tecan, AG, USA). For K562 cells, 100 μl of K562 cells (4 × 10^6^ cells/ml) were plated in 96-well plate and HHT concentration was adjusted to 0, 5, 10, 20, 40, 80, 160, 320, 640 nmol/L. The slight difference in HHT concentration chosen between K562 and U937 was to derive the best cytotoxicity curve for each cell line.

### Genome-wide SNP and expression data analysis

The genotyping and expression array data were obtained for all 278 LCLs and were quality controlled as previously described (Li et al., 2008; Niu et al., 2010; Jiang et al., 2013). These data are publicly available from the NCBI Gene Expression Omnibus (http://www.ncbi.nlm.nih.gov/geo) under Super Series accession numbers GSE24277 and GSE23120. Briefly, DNA from all the LCLs was genotyped using Illumina HumanHap 550K and 510S Beadchips, which assayed 561,303 and 487,407 SNPs, respectively. We also obtained publicly available Affymetrix SNP array 6.0 Chip SNP data involving 906,367 SNPs for the same cells to give us even better coverage. Quality control (QC) was performed for all of these SNPs prior to performing statistical analysis. Specifically, for data obtained with the Illumina 550K array, we removed 11,258 SNPs that had call rate <95%, 33,237 SNPs with minor allele frequencies (MAF) <5%, and 49 SNPs that deviated from Hardy-Weinberg equilibrium (HWE). Therefore, 516,759 Illumina 550K SNPs were used in the genome-wide SNP analysis. The same approach was used for the QC analysis of SNPs on the Illumina 510S platform which resulted in a total of 336,070 SNPs remained for analysis. QC analysis was also performed for the publicly available Affymetrix 6.0 SNPs. After removing 32,515 SNPs with call rate <95%, 133,553 SNPs with MAF <5%, and 324 SNPs that deviated from HWE, a total of 739,395 SNPs remained. After removing redundant SNPs genotyped on both Affy and Illumina platforms, 1,366,022 SNPs obtained from both platforms were available for the GWAS analysis. Genome-wide imputation was then performed in LCLs. Specifically, SNPs that were not genotyped but found in the multi-race (ALL) of the1000 genomes project (11/23/2010 released version) were imputed by race using BEAGLE v3.3.1 (Browning and Browning, 2007). SNPs in the 1000 genomes project with MAF <0.01 were excluded as well SNPs with BEAGLE imputation dosage R^2^ quality measure <0.3 were excluded from the further analysis, which resulted in a total of 5,384,559 imputed markers. Therefore, a total of 6,750,581 SNPs including imputed and genotyped were used in all the association studies described below.

For the expression array assays, total RNA was extracted from each of the cell lines using the RNeasy Mini Kit (QIAGEN Inc., Valencia, CA, USA). Total mRNA was assayed after hybridization to Affymetrix U133 plus 2.0 GenChips as previously described (Li et al., 2008; Niu et al., 2010). A total of 54,613 probe sets were used in the analysis.

### Transient transfection and RNA interference

SiRNAs for the candidate genes and negative control siRNA were purchased from QIAGEN (QIAGEN Inc., Valencia, CA, USA). 4–5 × 10^6^ cells were transfected with 500 nM of siRNA using Amaxa™ Cell Line Nucleofector™ transfection kit (Lonza, Koeln, Germany). After incubation overnight, cells were plated in 96-well plate for further drug treatment and MTS assay.

### Real-time quantitative reverse transcription PCR

Total RNA was isolated from cultured cells transfected with control or specific siRNA with the ZR RNA MiniPrep™ kit (Zymo Research, Irvine, CA, USA), followed by one-step qRT-PCR performed with SYBR® Green PCR Master Mix Kit (Applied Biosystem, Foster City, CA, USA). Specific primers for mRNA amplification were purchased from QIAGEN.

### Statistical analysis

#### Drug toxicity

The detailed description of analysis methods for accessing the association of cytotoxicity with SNP and/or mRNA expression data in these LCL cells has been described previously (Li et al., 2008; Niu et al., 2010; Jiang et al., 2013). Cytotoxicity phenotypes were determined by the best fitting curve using the R package “drc” (dose response curve) (http://cran.r-project.org/web/packages/drc.pdf) based on a logistic model. The AUC phenotype was determined using the best fitting curve by numerically determining the area under the estimated dose-response curve, from dose 0 to 320 nmol/L.

#### Association analysis

Partial correlations between: AUC and gene-expression; AUC and SNPs; and gene expression and SNPs were calculated; adjusting for sex, race, and variables addressing possible population stratification (sub-race variables). Partial correlations were then tested using *F*-tests, with multiple testing being addressed by *q*-values which control the false discovery rate (Storey, 2003). To construct the partial correlations, an adjusted AUC was constructed as the standardized residuals from a linear regression of Van der Waerden transformed AUC values regressed on gender, race and sub-race variables. Gene expression was first normalized using GCRMA (Ballman et al., 2004; Wu et al., 2004); log2 transformed normalized expression was then likewise regressed on gender, race and sub-race variables. SNP genotype was modeled as count of rare alleles, then regressed on gender, race and sub-race variables. Variables addressing possible population stratification (sub-race variables) were created from linkage disequilibrium thinned genome-wide SNPs using principle components analysis within each race (Price et al., 2006). The top 5 principle components were used within each race, thus analyses were adjusted for labeled cell line race and sub-racial features within each race (Li et al., 2008; Niu et al., 2010). For siRNA transfection experiments, group mean value for AUC and gene expression was compared by using Student's *t*-test.

#### Pathway analysis

For pathway analysis of top associated genes, we used Ingenuity Pathway Analysis (Ingenuity® Systems, www.ingenuity.com) for network analysis.

## Results

### HHT cytotoxicity

**Figure 1:**
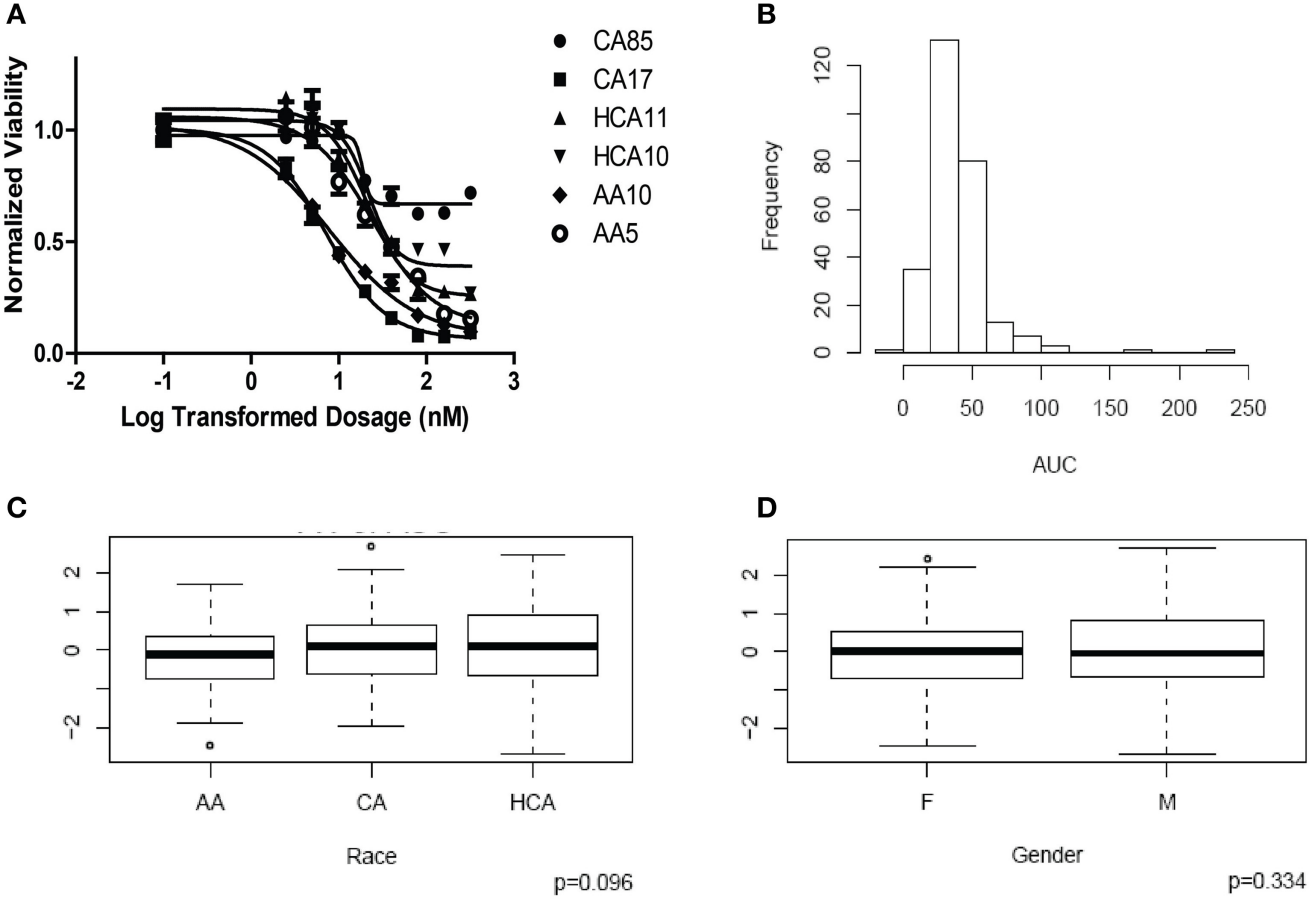
**Cytotoxicity of Homoharringtonine (HHT)**. **(A)** Representative cytotoxicity dose response curves for HHT. Two cell lines from each of the three ethnic groups (AA, Africa American; CA, Caucasian American and HC, Han Chinese American) were selected to illustrate a range of HHT cytotoxicity. The X-axis indicates the log transformed dosage (nM) and the y-axis indicates the cell viability normalized to control (without drug treatment). Symbols represent individual cell line from different ethnic groups. **(B)** Histograms of frequency distributions of AUC values for HHT. **(C)** Race effect on HHT cytotoxicity (AUC values). Y-axis represents normalized AUC values. **(D)** Gender effect on HHT cytotoxicity (AUC values). Y-axis represents normalized AUC values.

HHT cytotoxicity studies were performed to determine the range of variation in HHT AUC among the 278 individual LCL cell lines from three ethnic groups. Figure 1A shows representative HHT cytotoxicity data for a set of cell lines. The frequency distribution of AUC for HHT was shown in Figure 1B. AUC values did not show a significant difference among the three racial groups studied (*P* = 0.096, Figure 1C). Gender did not have a significant effect on AUC (*P* = 0.334, Figure 1D).

### Correlation between expression and AUC

**Figure 2:**
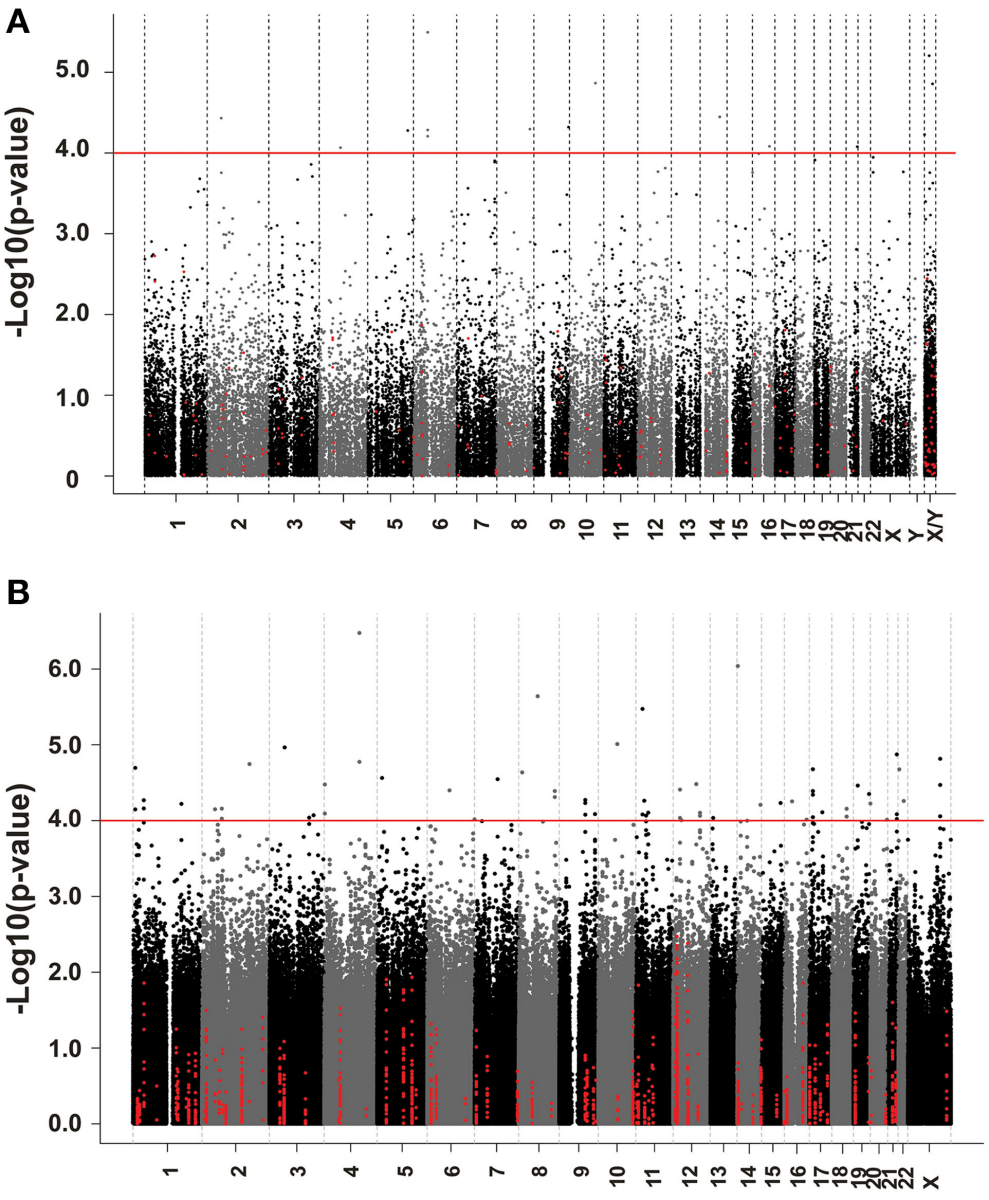
**Genome-wide association of mRNA expression and SNPs with HHT cytotoxicity**. **(A)** Association of basal gene expression with HHT AUC value for 278 LCL cell lines. The y-axis represents the –log_10_(*P*-value) for the association of individual expression array probe sets with HHT AUC, and the x-axis represents chromosomal location of expression probe sets. **(B)** Genome-wide SNPs association with HHT for 278 LCL cell lines. The y-axis represents the –log_10_(*P*-value) for the association of Genome-wide SNPs with HHT AUC, and the x-axis represents chromosomal location of SNPs. A *P*-value of 10^−4^ is highlighted with a red line.

We next performed correlation analysis for the association of expression array and HHT AUC data to identify genes which might be associated with HHT cytotoxicity at the gene expression level (Figure 2A). The association analysis identified 15 expression probe represented 10 annotated genes that were associated with HHT AUC with *P* < 10^−4^, and 96 individual probe sets represented 79 annotated genes with *P* < 10^−3^ (Supplementary Table S1). The most significant probe set for an annotated gene was *DST* (*P* = 3.2 × 10^−6^). Two other probe sets of this gene were also associated with HHT AUC (215016-x-at, *P* = 5.2 × 10^−5^; 212253-x-at, *P* = 6.19 × 10^−5^). *CTBP2* was found to have three expression probe sets associated with AUC value (*P* < 10^−4^) (201218-at, *P* = 6.24 × 10^−6^; 210835-s-at, *P* = 1.39 × 10^−5^; 201220-x-at, *P* = 5.92 × 10^−5^).

For the functional validation, we chose genes that had probe sets associated with AUC value with *P* < 10^−4^. Among these genes, we removed genes with low expression levels in the LCLs (<50 after GCRMA normalization). Therefore, three genes including *DST, CTBP2* and *CCDC88A* were selected for further functional studies (Table 1A). Furthermore, we subjected genes that were associated with AUC with *P* < 10^−4^ to a pathway based analysis using Ingenuity Pathway analysis tools. 5 gene signature networks that were significantly associated with the HHT were obtained (Table 2). The most significant network consists of 36 molecules or genes in network and 17/36 genes are associated with the functions such as cellular growth and proliferation, cell death and survival (Table 2). The IPA network provides graphical representation of the biological connections between genes as shown in **Figure 4**.

Table 1**Candidate gene selected for siRNA screening based on GWA analysis**.

**Table:** 

**A. mRNA Exp vs. AUC (PANEL 1)**					
**Gene symbol**	**Chr**.	**Probe id**	***P***	R^*^	Q^**^
DST	6	212254-S-at	3.20E–06	0.28	0.14
		215016-x-at	5.20E–05	0.24	0.24
		212253-x-at	6.19E–05	0.24	0.24
CTBP2	10	201218-at	6.24E–06	−0.27	0.14
		210835-s-at	1.39E–05	−0.26	0.16
		201220-x-at	5.92E–05	−0.24	0.24
DC88A	2	238759-at	3.71E–05	0.25	0.24
		221078_s_at	1.76E–04	0.29	0.23

**Table:** 

**B. SNP vs. AUC (PANEL 2)**							
**Gene symbol**	**Chr**.	**SNP**	**Position**	**Location**	MAF^***^	***P***	***R***
WDHD1/SOCS4	14	rs1187882	55488747	Intron of WDHD1 5′-upstream of SOCS4	0.15	5.07e–05	0.25
SOCS4/WDHD1	14	rs60805089	55503348	Intron of SOCS4 3′-downstream of WDHD1	0.24	9.73e–05	0.36
SOCS4	14	rs7150145	55508260	Intron	0.24	8.92e–05	0.36
ZNF638	2	rs2058899	71580129	Intron	0.37	6.94e–05	0.24
	2	rs17749748	71605145	Intron	0.38	7.70e–05	0.24
	2	rs12474072	71636036	Intron	0.38	7.78e–05	0.24
	2	rs6714975	71633389	Intron	0.38	9.42e–05	0.24
	2	rs4852782	71636240	Intron	0.38	9.42e–05	0.24
	2	rs6745907	71609073	Intron	0.38	9.66e–05	0.24

**Table:** 

**C. INTEGRATED ANALYSIs (PANEL 3)**													
**SNP**					**Genes**			**SNP vs. EXP**		**SNP vs. AUC**		**EXP vs. AUC**	
**SNP**	**Chr**.	**Closest gene**	**SNP location**	**MAF**	**Probe id**	**Chr**.	**Gene symbol**	***P***	R*	***P***	R*	***P***	R*
rs10771093	12	SOX5	Inton	0.214	212253_x_at	6	DST	5.34E-05	0.242	2.37E–05	0.258	3.20E–06	0.278
rs60342295	17	—	—	0.119	1566990_x_at	6	ARID1B	9.73E-05	−0.234	3.28E–05	−0.254	0.000811	−0.202
rs4531814	18	ZBTB7C	Inton	0.428	231325_at	8	UNC5D	0.00031	−0.217	7.95E–05	0.241	9.80E–06	−0.264
rs58707448	18	—	—	0.482	205352_at	3	SERPINI1	0.000138	0.229	4.60E–05	−0.249	6.96E–05	−0.238

* *Represents the correlation coefficient R-value for associations*.

** *Represents the false discovery rate Q-value*.

*** *Represents the minor allele frequency (MAF)*.

**Table 2:** **Top 5 networks associated with HHT response obtained by Ingenuity pathway analysis**.

**ID**	**molecules in network**	**Score**	**Focus molecules**	**Top diseases and functions**
1	AICDA, AKR1B1, Akt, ANKRD1, BANK1, CASP2, CASP12, caspase, CBS/LOC102724560, CCDC88A, CD3, Ck2, EBF1, ERK1/2, FADD, IgG1, Igm, Immun oglobulin, Insulin, Interferon alpha, Jnk, NAP1L1, NFkB (complex), NRF1, P38 MAPK, PAWR, PPIA, PRDX3, PRKCE, SCD5, SER PINB9, SIRT4, SPRY4, STX6, TRAF5	36	17	Cellular growth and proliferation, cell death and Survival, renal necrosis/cell death
2	ALG1L2, ATG9A, BRD3, C14orf2, CALM1 (includes others), CEP68, CHD4, CRYAA/LOC102724652, CTBP1, FAM178A, FXR2, GID8, GIMAP1, GIMAP7, GJA3, GON4L, HAGHL, ITFG1, LCMT1, MAEA, MAP1LC3B, MKLN1, MRPL44, MTMR14, OPTN, PPP2R4, PPP2R2B, PTEN, RAB30, RMND5A, SCCPDH, SMAD9, UBC, WHSC1L1, ZNF143	33	16	Ophthalmic disease, hereditary disorder, cellular Assembly and organization
3	APP, ARID1B, BAZ2B, C15orf39, CASP12, CDK3, CLIP4, COL17A1, CTBP2, CTPS2, DOCK4, DST, ELAVL1, FAM117B, FAM178A, FBXL2, FNDC3B, GATAD2A, GIMAP5, GIMAP6, IL13, ISCU, KIAA1549, KLF8, MOB1B, NEFH, PLXDC2, RAB33A, RAI2, RALGPS1, RASSF3, SH3BP5, TWISTNB, UBC, ZBTB20	33	16	Cellular compromise, neurological disease, Organismal injury and abnormalities
4	ACACA, AFF3, AKR1B1, APOBR, BCL3, CASP2, CLCN4, CMC4, COA4, COPS7B, DDIT3, EPO, FAHD2A, FOXO1, GNS, Hdac, HNF4A, IRF2BP2, ITGA6, KAT2B, MON1B, NRIP1, NTN1, NUDCD3, OCLN, ONECUT1, PPP1R15A, RASSF1, STK4, TEX10, TNF, UNC5D, UXT, VASH1, ZBTB45	28	14	Cell death and survival, renal necrosis/cell death, Lymphoid tissue structure and development
5	AChR, Akap9, CDH1, CREB1, DRD2, ERBB2, ESR1, G6PC, GCG, GPATCH2, HDAC2, IFNG, ITPR1, KIAA2022, LDL, NFKB1, NPY, phosphatase, PI3K(complex), PIAS1, PIAS3, Pka catalytic subunit, PLAT, POU5F1, PPP1CA, PPP1R15A, PPP3CB, PRKACA, PTEN, PTPRN, SERPINI1, SHIP, sphingomyelinase, SYNJ2, VCPIP1	8	5	Cellular compromise, neurological disease, Organismal injury and abnormalities

### Genome-wide SNP association with HHT AUC

We performed an analysis of the association of genome-wide SNPs with HHT AUC (Figure 2B). A total of 561,303 SNPs on the Illumina 550K SNP array and 487,044 SNPs on the Illumina 510S SNP array had been genotyped using DNA from each of these 278 cell lines. We also had access to publicly available Affymetrix 6.0 SNP array data. After quality control and genome wide imputation, a total of 6,750,581 SNPs were used in the association studies.

GWAS was performed between SNPs and HHT AUC values. Although none of the SNPs reached genome-wide significance (*P* < 10^−8^), 18 SNPs had *P* < 10^−5^ and 281 SNPs had *P* < 10^−4^ (Supplementary Table S2). The *P* value for the most significant SNPs (rs1250991) was 3.1 × 10^−7^.

For functional validation, we identified 15 regions/loci that contained at least two SNPs with *P* < 10^−4^ within 50 kb in each of these regions (a total of 95 SNPs). We defined these regions as “SNP peak region.” From these regions, we picked up three genes which were also expressed in LCLs and leukemia cells for further analysis (Table 1B).

### Integrated analysis

The effect of genetic variation on HHT-induced cytotoxicity might result in part from the regulation of gene expression. Therefore, we performed an integrated analysis that included data from SNPs, basal expression, and HHT AUC, as reported before (Li et al., 2009; Niu et al., 2010; Jiang et al., 2013).

Specifically, for the top HHT associated SNPs, (i.e., SNP with *P* < 10^−4^), we determined their association with gene expression using *P* < 10^−4^ as a cutoff. These SNP–associated genes were then narrowed down to those whose mRNA gene expression probe sets that were also associated with HHT cytotoxicity (*P* < 10^−3^). We used the less stringent criteria to capture more potential candidate genes for further functional validation. Through this integrated analysis, we identified 4 unique SNPs that were associated with expression and AUC (Table 1C). None of the SNPs were in cis-regulatory region.

### Functional validation of candidate genes in human leukemia cells

**Figure 3:**
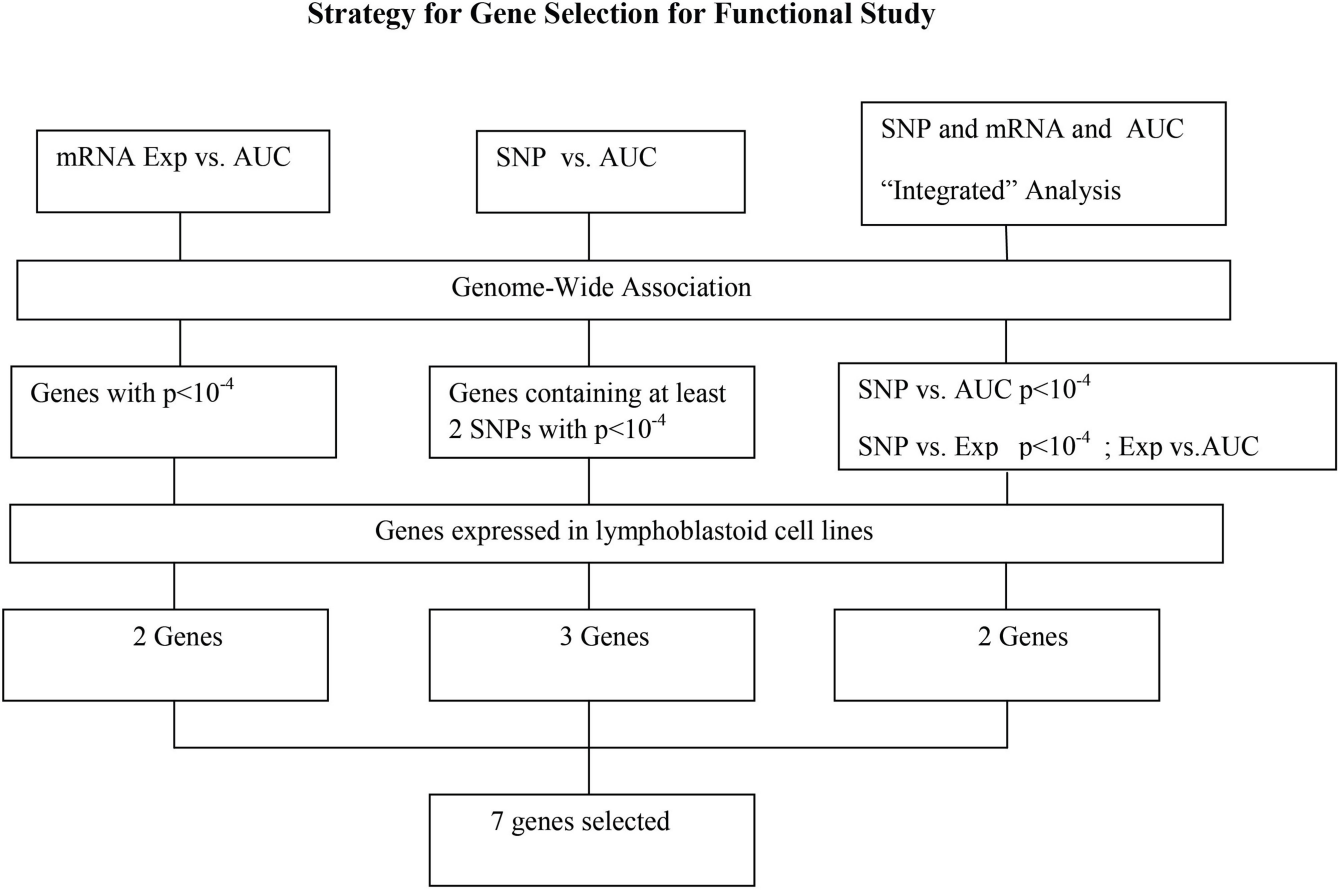
**Schematic diagram of the strategy for selecting candidate genes for functional validation**. A total of 13 candidate genes were selected based on genome-wide association of expression (Exp) vs. AUC, SNP vs. AUC and “Integrated” analysis, as described in the text. After removing those that were not expressed in our LCLs, 7 genes were further selected for functional validation.

In summary, we selected 7 genes based on the strategy shown in Figure 3 to perform further functional validation in human leukemia cell lines: U937 and K562. Since HHT is commonly used in the patients of AML and CML, leukemic monocyte cell line U937 and human erythromyeloblastoid leukemia cell line K562 cells were used for further studies. These functional experiments involved siRNA knockdown, followed by MTS cytotoxicity. Knocking down *CCDC88A, CTBP2*, and *SOCS4* genes exhibited significant resistance to HHT sensitivity in U937 and K562 cells (Figure 5). The others did not show significantly impact on HHT sensitivity.

**Figure 4:**
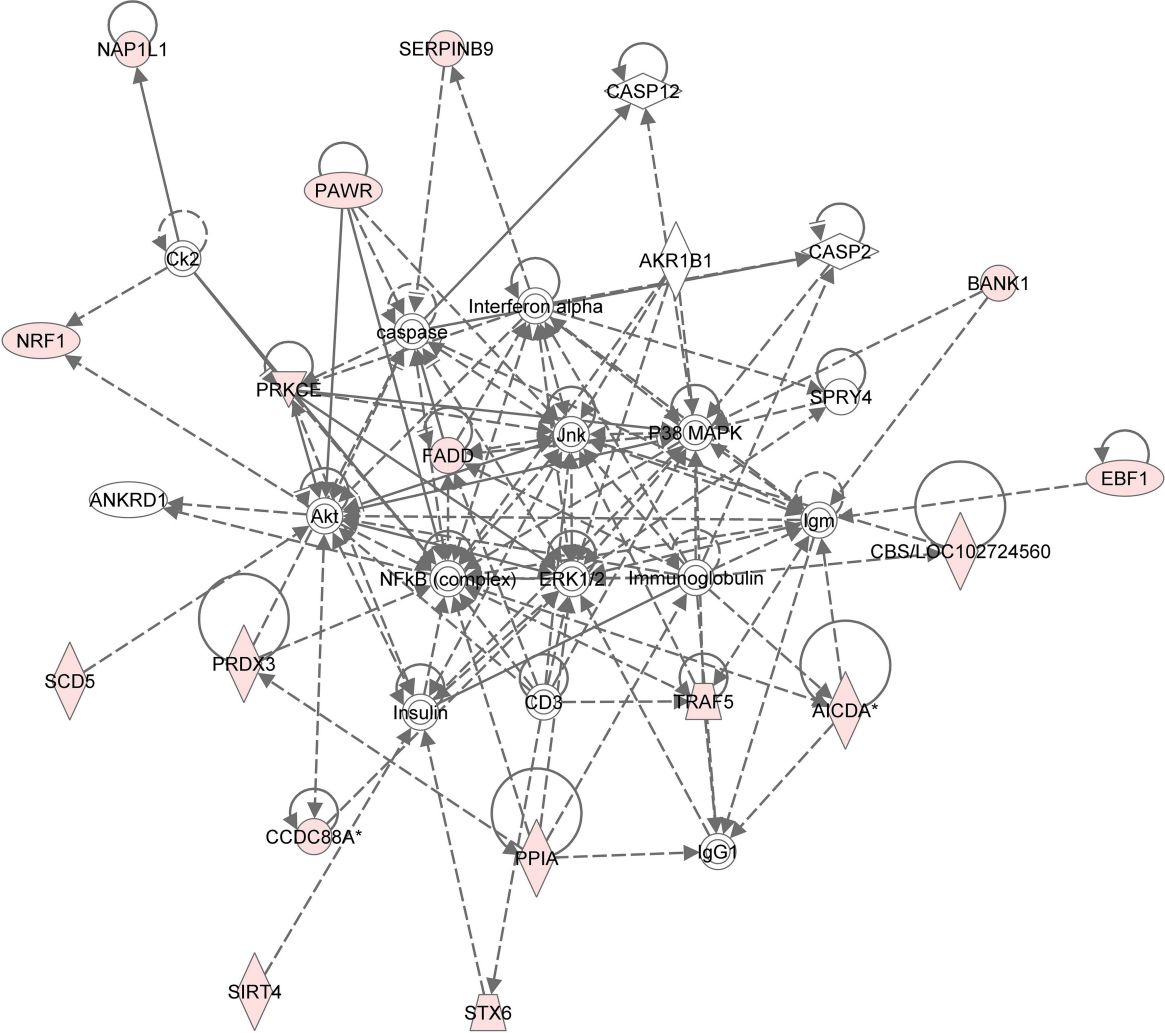
**Gene network analysis of top HHT associated genes**. Ingenuity pathway analysis was performed. Each node in the network represents a gene and edge represents a relationship, the pink colored nodes in the network are the significant genes that are correlated with HHT AUC phenotype.

**Figure 5:**
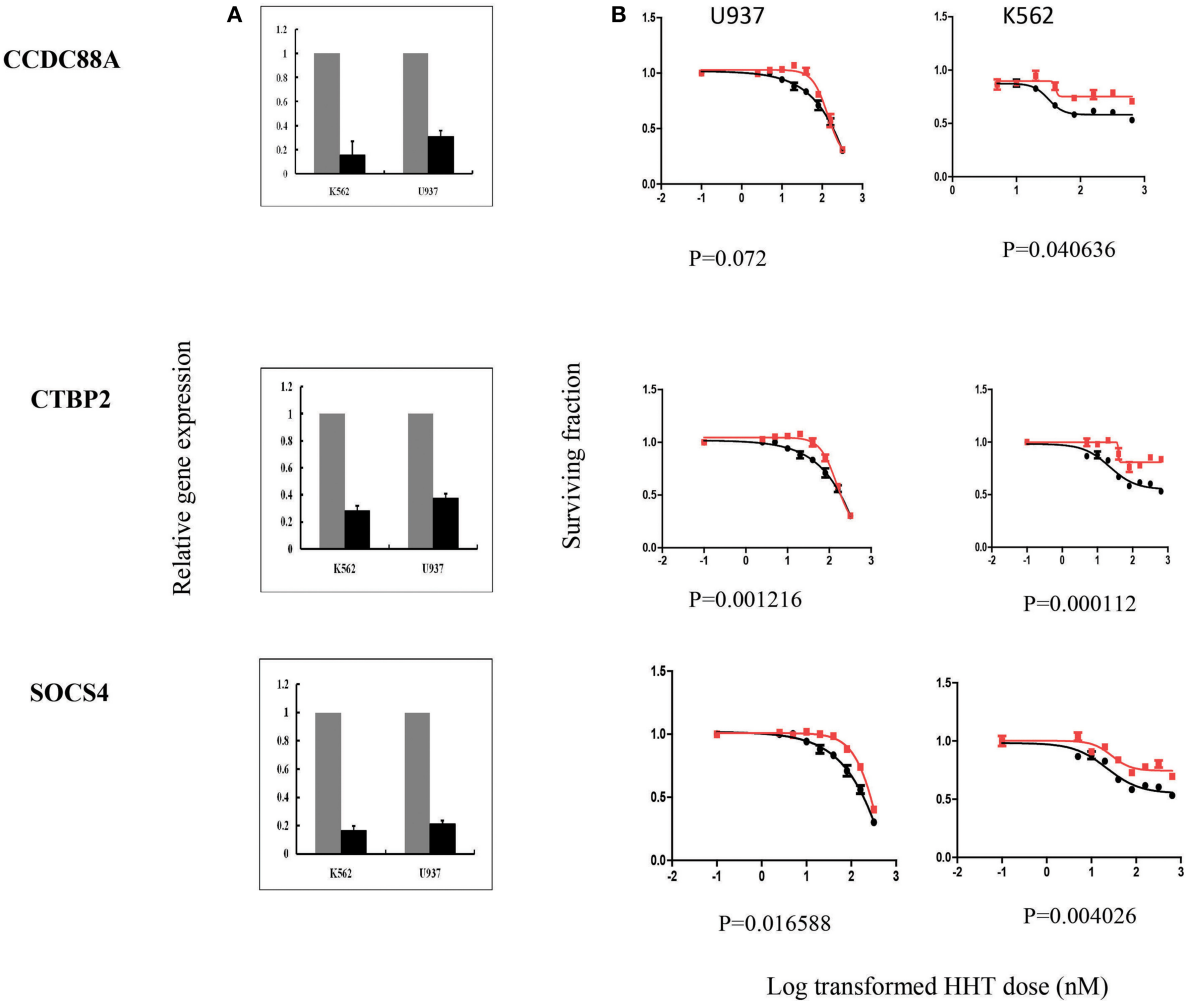
**SiRNA screening of candidate genes by MTS assay in leukemia cell lines**. Data are shown for 3 of the 7 candidate genes that were studied in U937, K562 leukemia cell lines by MTS assay after siRNA knockdown. Experiments were repeated in triplicate with at least two independent experiments. Error bars indicate standard error of the mean (SEM) values. Significance was defined by *P*-values. **(A)** knockdown efficiency was determined by qRT-PCR. The y-axis indicates relative gene expression after siRNA knockdown when compared with negative control siRNA. **(B)** MTS assays. The x-axis indicates the log transformed HHT dose, and the y-axis indicates the surviving fraction after exposure to HHT.

Taken together, through the initial screening studies in LCLs and further functional validation in leukemia cell lines, we found that the expression of three genes, *CCDC88A, CTBP2*, and *SOCS4*, were involved in HHT-induced response.

## Discussion

HHT is a commonly used drug for the treatment of acute and chronic myeloid leukemia in China. Recently it has also been approved by FDA for the treatment of CML resistant to TKIs. The addition of HHT improved both CR rate and Relapse Free Survival (RFS) rate. However, its major dose-limiting toxicity is bone marrow suppression. The response to HHT varied widely as some patients showed no response whereas the bone-marrow suppression was severe. Therefore, a better understanding of the biology underlying the variation to HHT could help us maximize HHT efficacy while minimizing drug related toxicity.

Recent studies have raised the possibility that genetic variation might contribute to individual variation in drug response. Many pharmacogenomic studies have successfully used LCL models to illustrate the contribution of germline genetic variations to variation in chemotherapeutic drug response. For example, using the LCL system, previous findings have identified potential biomarkers for response to a series of drugs including Ara-C, 5-FU, Asparaginase, Daunorubicin (Watters et al., 2004; Huang et al., 2007; Peters et al., 2009; Chen et al., 2011). In some cases, findings from the LCLs were further validated in clinical studies. *FKBP5*, which was found as a biomarker of Ara-C response using the LCL system, was further studied at mechanistic levels showing that FKBP5 promoted the dephosphorylation of AKT and downregulated the AKT activity, which in turn, increased cytotoxicity by triggering apoptosis in the presence of Ara-C (Pei et al., 2009). These findings were validated in a clinical study, in which SNPs within *FKBP5* were genotyped in a cohort of 187 pediatric acute myeloid leukemia patients treated with Ara-C. Two of the SNPs were associated with both event-free and overall survival (Mitra et al., 2011). Based on the potential impact of these findings in individualizing chemotherapy, we aimed to use this well-established LCLs model to find biomarkers responsible for HHT cytotoxicity.

In the present study, we performed a genome-wide association analysis using 278 LCLs for which we had 1.3 million SNPs, basal gene expression data, and HHT cytotoxicity AUC phenotype to identify genes which might be responsible for HHT sensitivity. Based on these data, our study identified top HHT cytotoxicity associated gene networks (Figure 4 and Table 2) that might help generate hypothesis as well as yield a total of 7 genes which were also expressed in LCLs and leukemia cell lines for further studies. The LCL system used in our screening studies has limitations. EBV transformation might cause chromosomal instability and the cellular changes in LCLs (Sie et al., 2009), and other factors such as cell growth rate and ATP level can also have effect on cytotoxicity (Choy et al., 2008). Since these LCL cell lines may not represent the response of leukemia cell lines to HHT, we identified 7 top candidate genes to perform functional validation studies using siRNA knockdown in two leukemia cell lines: U937 and K562 cells. U937 cells lines are derived from leukemic monocyte cells and K562 cells are also one of myelogenous leukemia cell lines. These two cell lines, tended to show similar results for the three genes (*CCDC88A, CTBP2, SOCS4*) tested. Our results suggested that knockdown of these genes made cells more resistant to HHT.

*CCDC88A*, also named as Coiled-Coil Domain Containing 88A, encodes a member of the Girdin family of coiled-coil domain containing proteins. The encoded protein enhances Akt signaling by mediating phosphoinositide 3-kinase (PI3K)-dependent activation of Akt. AKT activation regulates DNA replication and cell proliferation by phosphorylating the downstream effectors GSK3 and FOXO1/FKHR. The PI3K/AKT and mTOR signaling pathways are activated in acute myeloid leukemia (Park et al., 2010). Although the mechanism responsible for CCDC88A involvement in HHT response is unclear, given the roles of AKT in the development of leukemia, it was not surprising that this gene might play a role in variable response to HHT. However, we recognized that the CCDC88A had a positive association with HHT cytotoxicity in LCLs, while our knockdown showed the opposite effect, which might be due to the difference between LCLs and Leukemia cell lines. Further mechanistic studies will be needed to confirm the results. The protein encoded by *SOCS4* belongs to the suppressor of cytokine signaling (SOCS), also known as STAT-induced STAT inhibitor (SSI), protein family. SOCS family members are known to be cytokine-inducible negative regulators of cytokine signaling, including JAK1/STAT3 pathway. SOCS4 has been also demonstrated to regulate EGFR signaling *in vitro* (Chan et al., 2009). Therefore, regulation of both pathways could contribute to HHT cytotoxicity. *CTBP2* gene produces alternative transcripts encoding two distinct proteins. One protein is a transcriptional repressor, while the other isoform is a major component of specialized synapses known as synaptic ribbons. Transcription corepressor CTBP2 has been reported to directly bind acinus, which is regulated by NGF (nerve growth factor), inhibiting its stimulatory effect on cyclin A1 expression in leukemia (Trengove and Ward, 2013). Although there is limited information with regard to the involvement of these genes to HHT response, our results suggest a possible role for their relationship with variation in HHT response. Further mechanistic studies of these genes as well as additional association studies in patients treated with HHT are needed to validate the genes as biomarkers of HHT response.

## Conclusions

In summary, we used a pharmacogenomics approach based on the use of genomic data rich LCLs system to identify genetic candidate that might contribute to HHT cytotoxicity. Functional validation of these candidate genes may further support the feasibility of utilizing these genes for clinical validation of HHT response. These results may enhance our ability to individualized treatment with HHT.

## Author contributions

Yin Tong and Liewei Wang designed the study and wrote the manuscript. Yin Tong performed the experiments. Gregory Jenkins and Anthony Batzler performed the statistical analyses. All the authors read, revised the draft manuscript and approved the final version.

## Conflict of interest statement

The authors declare that the research was conducted in the absence of any commercial or financial relationships that could be construed as a potential conflict of interest.
