# Supplementary material for: Identification of genetic variants or genes that are associated with Homoharringtonine (HHT) response through a genome-wide association study in human lymphoblastoid cell lines (LCLs)
Source: Front Genet. 2015 Jan 13;5:465. doi: 10.3389/fgene.2014.00465 (PMC4292778; doi:10.3389/fgene.2014.00465)
Supplement: Supplementary file 1 [file Table1.PDF]

	probe.id	chromosome	P value	Bonferroni corrected P value	r	n	Gene
1	212254_s_	6	3.20E-06	0.175	0.278	272	DST
2	201218_at	NA	6.24E-06	0.341	-0.27	272	CTBP2
3	203482_at	10	1.36E-05	0.745	-0.26	272	FAM178A
4	210835_s_	NA	1.39E-05	0.759	-0.26	272	CTBP2
5	203940_s_	14	3.57E-05	1	-0.248	272	VASH1
6	238759_at	2	3.71E-05	1	0.247	272	CCDC88A
7	212547_at	9	4.78E-05	1	-0.244	272	BRD3
8	1553849_a	8	5.06E-05	1	-0.243	272	CCDC26
9	215016_x_	6	5.20E-05	1	0.243	272	DST
10	233261_at	5	5.27E-05	1	-0.243	272	EBF1
11	201220_x_	NA	5.92E-05	1	-0.241	272	CTBP2
12	212253_x_	6	6.19E-05	1	0.24	272	DST
13	240676_at	16	8.28E-05	1	-0.236	272	---
14	1553972_a	21	8.35E-05	1	-0.236	272	CBS
15	224018_s_	4	8.61E-05	1	0.236	272	SCD5
16	221515_s_	16	0.000102	1	-0.233	272	LCMT1
17	214769_at	23	0.000113	1	-0.232	272	CLCN4
18	240393_at	19	0.000122	1	0.231	272	---
19	218805_at	7	0.000125	1	-0.231	272	GIMAP5
20	229367_s_	7	0.00013	1	-0.23	272	GIMAP6
21	205352_at	3	0.000138	1	0.229	272	SERPINI1
22	209075_s_	12	0.000154	1	0.228	272	ISCU
23	204004_at	12	0.000171	1	-0.226	272	PAWR
24	206039_at	23	0.000171	1	-0.226	272	RAB33A
25	217556_at	23	0.000173	1	-0.226	272	CLCN4
26	223485_at	16	0.000174	1	-0.226	272	HAGHL
27	235621_at	NA	0.000175	1	0.226	272	FAHD2A /// LOC285014
28	221078_s_	2	0.000176	1	0.226	272	CCDC88A
29	225032_at	3	0.000195	1	0.224	272	FNDC3B
30	236026_at	1	0.000209	1	-0.223	272	GPATCH2
31	213156_at	3	0.000214	1	0.223	272	---
32	243009_at	NA	0.000236	1	-0.221	272	---
33	201270_x_	7	0.000273	1	-0.219	272	NUDCD3
34	215610_at	NA	0.000277	1	0.219	272	---
35	224571_at	1	0.000281	1	-0.219	272	IRF2BP2
36	204352_at	1	3.00E-04	1	-0.218	272	TRAF5
37	231325_at	8	0.00031	1	-0.217	272	UNC5D
38	212335_at	12	0.000313	1	0.217	272	GNS
39	208590_x_	13	0.000322	1	0.217	272	GJA3
40	210552_s_	9	0.000328	1	0.216	272	RALGPS1
41	244791_at	13	0.000328	1	-0.216	272	---
42	219777_at	7	0.00037	1	-0.214	272	GIMAP6
43	205003_at	7	0.000382	1	-0.214	272	DOCK4
44	1556442_x	NA	4.00E-04	1	0.213	272	---
45	226431_at	2	0.000404	1	-0.213	272	FAM117B
46	1552316_a	7	0.000405	1	-0.213	272	GIMAP1
47	201826_s_	1	0.000442	1	-0.212	272	SCCPDH
48	244041_at	1	0.000471	1	-0.211	272	---
49	212675_s_	2	0.00048	1	-0.21	272	CEP68
50	221449_s_	16	0.00049	1	0.21	272	ITFG1
51	243189_at	7	0.000525	1	-0.209	272	---
52	241679_at	6	0.000529	1	-0.209	272	---
53	216862_s_	23	0.000563	1	-0.208	272	MTCP1NB
54	226336_at	7	0.000579	1	-0.207	272	PPIA
55	226784_at	7	0.000582	1	-0.207	272	TWISTNB
56	233771_at	5	0.000582	1	-0.207	272	---
57	64064_at	7	0.000587	1	-0.207	272	GIMAP5
58	201219_at	NA	0.000588	1	-0.207	272	CTBP2
59	219667_s_	4	0.00059	1	-0.207	272	BANK1
60	220047_at	12	0.00061	1	-0.207	272	SIRT4

61	220647_s_	11	0.000612	1	-0.206	272	CHCHD8
62	210612_s_	6	0.000614	1	-0.206	272	SYNJ2
63	241475_at	8	0.000635	1	-0.206	272	BREA2
64	227198_at	2	0.000649	1	-0.206	272	AFF3
65	242814_at	6	0.000652	1	0.205	272	SERPINB9
66	223555_at	NA	0.00066	1	0.205	272	GON4L
67	220023_at	16	0.000667	1	-0.205	272	APOB48R
68	233378_at	5	0.000676	1	0.205	272	---
69	239105_at	14	0.000692	1	0.205	272	---
70	244076_at	23	0.000702	1	-0.204	272	KIAA2022
71	202279_at	14	0.000714	1	-0.204	272	C14orf2
72	208754_s_	12	0.000729	1	-0.204	272	NAP1L1
73	226101_at	2	0.000729	1	-0.204	272	PRKCE
74	226250_at	3	0.000736	1	0.204	272	---
75	1558458_a	7	0.000756	1	0.203	272	LOC401320
76	218104_at	9	0.000773	1	-0.203	272	TEX10
77	209766_at	10	0.000774	1	0.203	272	---
78	224499_s_	12	0.000786	1	-0.202	272	AICDA
79	214436_at	3	0.000791	1	-0.202	272	FBXL2
80	1559151_a	15	0.000806	1	-0.202	272	---
81	219841_at	12	0.000808	1	-0.202	272	AICDA
82	225696_at	2	0.000811	1	-0.202	272	COPS7B
83	238043_at	6	0.000811	1	-0.202	272	ARID1B
84	202535_at	11	0.000851	1	-0.201	272	FADD
85	228071_at	7	0.000852	1	-0.201	272	GIMAP7
86	222143_s_	3	0.000854	1	-0.201	272	MTMR14
87	227775_at	15	0.000865	1	-0.201	272	CELF6
88	234294_x_	19	0.000904	1	-0.2	272	GATAD2A
89	228003_at	11	0.000908	1	0.2	272	RAB30
90	227116_at	16	0.000915	1	-0.2	272	MON1B
91	233596_at	5	0.000955	1	-0.199	272	---
92	212482_at	2	0.000962	1	-0.199	272	RMND5A
93	234988_at	8	0.000965	1	-0.199	272	VCPIP1
94	211796_s_	7	0.000984	1	-0.199	272	TRBC1 /// TRBC2
95	1562677_a	5	0.001	1	-0.198	272	---
96	224971_at	2	0.001	1	-0.198	272	MRPL30

Supplementary Table S1. Expression probes that were assoicated with HHT AUC values with p<10E-3
