# Supplementary material for: Identification of genetic variants or genes that are associated with Homoharringtonine (HHT) response through a genome-wide association study in human lymphoblastoid cell lines (LCLs)
Source: Front Genet. 2015 Jan 13;5:465. doi: 10.3389/fgene.2014.00465 (PMC4292778; doi:10.3389/fgene.2014.00465)
Supplement: Supplementary file 2 [file Table2.PDF]

SNP	chromosome	Position	MAF	Minor allele	genotype*	R-value	P-value	Closest Gene
rs12509991	4	126996870	0.353	T	I	0.31	3.18E-07	
rs10518485	4	126996974	0.354	A	O	0.3096	3.34E-07	
rs4288020	4	126999851	0.327	T	I	0.301	6.65E-07	
rs9355211	6	168816452	0.112	T	I	0.301	6.83E-07	
rs279875	9	998869	0.296	A	I	-0.301	7.14E-07	DMRT3
rs1263806	14	21982957	0.301	G	O	0.2984	9.12E-07	SALL2/METTL3
rs4382051	4	127001808	0.332	G	I	0.296	1.04E-06	
rs7461360	8	68678150	0.301	C	O	0.2872	2.29E-06	
rs7106204	11	24236096	0.208	C	O	-0.2826	3.36E-06	
rs67383251	9	137171766	0.209	C	I	-0.276	6.01E-06	
rs4769475	13	26731714	0.237	T	I	-0.275	6.12E-06	
c9.93990003.b37p0	9	93990003	0.117	C	I	0.274	6.70E-06	AUH
rs12514883	5	17791091	0.282	G	I	-0.273	7.15E-06	
rs7332725	13	26732262	0.239	G	I	-0.273	7.62E-06	
rs4769474	13	26731453	0.24	A	I	-0.272	7.90E-06	
rs4769473	13	26731419	0.24	A	I	-0.272	7.93E-06	
rs12498791	4	126997254	0.301	C	I	0.272	8.06E-06	
rs12218365	10	68576077	0.227	G	O	0.2699	9.76E-06	CTNNA3
rs358798	3	55325767	0.269	A	O	-0.2691	1.08E-05	
rs3135134	4	2353309	0.245	T	I	0.267	1.14E-05	ZFYVE28
rs6645941	23	116944598	0.399	C	I	-0.266	1.30E-05	
rs6645395	23	116926709	0.404	T	I	-0.266	1.32E-05	
rs6645939	23	116944101	0.4	C	I	-0.265	1.33E-05	
rs1044317	21	43716901	0.454	A	O	0.2659	1.34E-05	ABCG1
rs62577453	9	137171576	0.237	C	I	-0.265	1.40E-05	
rs12276488	11	24250211	0.206	A	I	-0.265	1.42E-05	
rs16837123	2	206121666	0.13	A	I	-0.265	1.43E-05	PARD3B
rs113326972	2	20713008	0.135	C	I	-0.264	1.47E-05	
rs6645940	23	116944353	0.395	T	I	-0.264	1.47E-05	
rs5956798	23	116922512	0.384	G	O	-0.2637	1.53E-05	
rs2978299	8	30596834	0.405	G	I	-0.263	1.56E-05	UBXN8
cX.116963778.b37p0	23	116963778	0.373	C	I	-0.263	1.59E-05	
rs1517822	12	83531782	0.309	A	I	0.263	1.63E-05	TMTC2
rs12647698	4	126990576	0.279	A	O	0.2625	1.68E-05	
rs6131711	20	15776418	0.444	A	I	0.262	1.72E-05	MACROD2
rs2164963	2	171323416	0.213	C	O	0.2636	1.79E-05	MYO3B
cX.116963716.b37p0	23	116963716	0.38	A	I	-0.261	1.85E-05	
rs11654789	17	16772110	0.132	A	I	-0.261	1.86E-05	
rs10864350	1	8394796	0.381	G	O	0.2601	2.01E-05	SLC45A1
rs10991866	9	94036674	0.082	G	I	0.26	2.04E-05	AUH
rs16946773	17	12800304	0.446	A	O	0.26	2.10E-05	ARHGAP44
rs9913399	17	12784368	0.175	G	I	0.26	2.10E-05	ARHGAP44
rs854961	22	20278004	0.387	G	O	-0.26	2.11E-05	LOC440792
rs358801	3	55328378	0.267	A	I	-0.259	2.12E-05	
rs13185066	5	17792338	0.289	T	I	-0.259	2.22E-05	
rs12610429	19	48905145	0.087	A	I	-0.259	2.24E-05	GRIN2D
rs11630766	15	54586473	0.381	A	I	-0.259	2.25E-05	UNC13C
rs6981317	8	12695190	0.399	A	O	0.2588	2.31E-05	
rs10991851	9	94022836	0.083	A	I	0.258	2.31E-05	AUH
rs6479332	9	93896652	0.314	A	I	-0.258	2.35E-05	LOC100128909
rs10771093	12	24696495	0.214	A	I	0.258	2.37E-05	SOX5
rs55818909	2	163508071	0.166	T	I	-0.258	2.39E-05	KCNH7
c17.16770919.b37p0	17	16770919	0.162	T	I	-0.257	2.52E-05	
rs76598447	9	94031690	0.125	C	I	0.257	2.56E-05	AUH
rs2568413	8	130421811	0.291	A	I	0.256	2.73E-05	
rs10059062	5	17793212	0.287	G	O	-0.256	2.74E-05	
rs71580652	5	25028918	0.378	A	I	-0.256	2.82E-05	
rs79413176	9	94066964	0.129	C	I	0.256	2.82E-05	AUH
rs2579877	8	130421371	0.29	G	I	0.256	2.83E-05	
rs1860610	7	83149564	0.217	C	O	0.257	2.84E-05	SEMA3E
rs2630512	8	130420749	0.29	T	I	0.256	2.84E-05	
rs2719222	8	130421932	0.29	G	I	0.255	2.85E-05	
rs61916118	11	129707902	0.258	A	I	-0.255	2.85E-05	TMEM45B
rs1827522	3	55330190	0.262	C	I	-0.255	2.86E-05	
rs12525206	6	168816823	0.147	A	I	0.255	2.92E-05	
rs28420974	9	101083419	0.136	C	I	-0.255	2.92E-05	GABBR2
rs4148140	21	43718611	0.47	T	I	0.255	2.92E-05	ABCG1
rs12952427	17	16775721	0.134	A	I	-0.255	3.04E-05	
rs3135133	4	2353467	0.229	A	I	0.255	3.05E-05	ZFYVE28
rs358796	3	55324445	0.262	T	I	-0.255	3.06E-05	
rs2568408	8	130415010	0.29	A	I	0.254	3.10E-05	
rs2109543	7	83152022	0.222	G	I	0.254	3.16E-05	SEMA3E
rs77165341	9	93973165	0.125	T	I	0.254	3.24E-05	AUH/FP2234
rs2108541	17	12796796	0.172	A	I	0.254	3.28E-05	ARHGAP44
rs60342295	17	16773460	0.119	T	I	-0.254	3.28E-05	
rs1517821	12	83531453	0.307	A	O	0.2549	3.30E-05	TMTC2
rs35715519	5	36436537	0.107	A	I	0.253	3.31E-05	
rs3128832	4	2353834	0.312	A	O	0.2538	3.34E-05	ZFYVE28
rs11080229	17	31919099	0.188	G	I	-0.253	3.35E-05	ASIC2
rs6645400	23	116970152	0.368	G	O	-0.2546	3.38E-05	
rs34843293	17	16777627	0.138	C	I	-0.253	3.39E-05	
rs2322657	17	12792948	0.172	T	I	0.253	3.43E-05	ARHGAP44

rs6512042	19	15625172	0.11	A	O	-0.2529	3.45E-05	CYP4F22/LOC100422106
rs72895155	18	37512417	0.227	A	I	0.253	3.45E-05	
c9.93990974.b37p0	9	93990974	0.128	A	I	0.253	3.48E-05	AUH
rs2016932	8	130416567	0.29	C	I	0.253	3.48E-05	
rs358802	3	55328415	0.385	C	I	-0.253	3.53E-05	
rs12844666	23	116971438	0.472	G	I	-0.252	3.67E-05	
rs11651914	17	16776999	0.135	A	I	-0.252	3.68E-05	
rs12313344	12	34855158	0.149	A	I	-0.252	3.71E-05	
rs11221871	11	129708766	0.255	G	I	-0.252	3.72E-05	TMEM45B
rs4791518	17	12798730	0.171	A	I	0.252	3.72E-05	ARHGAP44
rs55909109	17	31917991	0.188	A	I	-0.252	3.80E-05	ASIC2
rs12311778	12	24701279	0.199	A	O	0.2512	3.90E-05	SOX5
rs59919391	15	101096789	0.156	C	I	-0.251	3.92E-05	PRKXP1
rs59992343	11	129710515	0.255	T	I	-0.251	3.96E-05	TMEM45B
rs2746313	6	80366145	0.306	G	O	0.2514	3.99E-05	SH3BGRL2
rs11078095	17	12797600	0.171	T	I	0.251	4.04E-05	ARHGAP44
rs11700907	21	43709666	0.253	C	I	-0.251	4.04E-05	ABCG1
rs12504181	4	77145441	0.204	A	I	0.251	4.06E-05	
rs11078094	17	12797461	0.171	G	I	0.251	4.07E-05	ARHGAP44
rs7221845	17	12793942	0.171	T	O	0.2507	4.07E-05	ARHGAP44
rs2719194	8	130417371	0.289	G	O	0.2525	4.07E-05	
rs6645952	23	116971169	0.376	G	I	-0.251	4.08E-05	
rs7021269	9	94090842	0.125	T	I	0.25	4.16E-05	AUH
rs79883029	9	94088054	0.125	T	I	0.25	4.16E-05	AUH
rs7035286	9	94081913	0.125	A	I	0.25	4.21E-05	AUH
rs10991891	9	94097726	0.125	T	I	0.25	4.23E-05	AUH
rs72895171	18	37519937	0.227	C	I	0.25	4.25E-05	
cX.116965986.b37p0	23	116965986	0.418	C	I	-0.25	4.37E-05	
rs3135130	4	2357680	0.222	T	I	0.25	4.37E-05	ZFYVE28
rs10412597	19	56473189	0.256	A	O	0.2504	4.45E-05	NLRP8
rs10991873	9	94062924	0.125	G	I	0.249	4.54E-05	AUH
rs10991905	9	94124912	0.126	A	I	0.249	4.54E-05	AUH
rs12949676	17	12784811	0.173	A	O	0.2496	4.55E-05	ARHGAP44
rs58707448	18	53585102	0.482	T	I	-0.249	4.60E-05	
rs1278910	14	32404616	0.482	T	I	0.249	4.71E-05	LOC100506110
rs76284073	9	94102902	0.125	G	I	0.249	4.73E-05	AUH
rs78973481	9	93962422	0.125	T	I	0.249	4.74E-05	FP2234
rs1278908	14	32404172	0.482	G	I	0.249	4.75E-05	LOC100506110
rs1278909	14	32404207	0.482	G	I	0.249	4.75E-05	LOC100506110
rs7695543	4	77175108	0.258	A	I	0.248	4.88E-05	FAM47E
rs2579875	8	130421433	0.291	G	O	0.2491	4.89E-05	
rs9668359	12	34824882	0.162	C	I	-0.248	4.93E-05	
rs71371199	17	16766947	0.166	G	I	-0.248	5.05E-05	COTL1P1
rs60299719	15	101096752	0.154	T	I	-0.248	5.06E-05	PRKXP1
rs1187882	14	55488747	0.336	C	I	0.248	5.07E-05	WDHD1/SOCS4
rs61913682	11	129711822	0.254	T	I	-0.248	5.11E-05	TMEM45B
rs8134778	21	43725617	0.431	T	I	0.248	5.11E-05	TFF3/ABCG1
rs9855124	3	187174310	0.403	A	I	-0.248	5.11E-05	
rs603005	1	39205993	0.204	T	I	-0.248	5.12E-05	
rs6645394	23	116914931	0.35	G	I	-0.247	5.15E-05	
rs10991898	9	94118258	0.127	A	O	0.2469	5.34E-05	AUH
rs665984	1	39203534	0.204	T	O	-0.2468	5.38E-05	
rs679737	1	39203648	0.204	C	I	-0.247	5.39E-05	
rs11502506	12	34840528	0.15	A	I	-0.247	5.41E-05	
rs12580382	12	34847880	0.175	A	I	-0.247	5.45E-05	
rs72895162	18	37515434	0.216	G	I	0.247	5.45E-05	
rs78737354	4	77136858	0.164	T	I	0.247	5.45E-05	SCARB2
rs3781852	11	30600383	0.289	A	O	0.2509	5.46E-05	MPPED2
rs5756080	22	36527967	0.319	G	O	0.2474	5.51E-05	APOL3
rs12587038	14	28782266	0.247	G	I	-0.246	5.51E-05	
rs1615164	16	27860811	0.48	C	O	-0.2463	5.57E-05	GSG1L
rs62172744	2	206113141	0.108	T	I	-0.246	5.60E-05	PARD3B
rs7700261	5	32632839	0.249	T	I	0.246	5.82E-05	
rs11073770	15	88820515	0.306	C	O	-0.2461	5.85E-05	LOC283738
rs10820842	9	93996118	0.142	G	O	0.2456	5.86E-05	AUH
rs61965278	13	75230166	0.347	G	I	-0.246	5.90E-05	
rs1410163	13	75229399	0.333	T	I	-0.245	5.92E-05	
rs6035505	20	259156	0.375	A	O	-0.2454	5.95E-05	C20orf96
rs2839483	21	43721037	0.459	G	I	0.245	5.97E-05	ABCG1
rs1023156	21	43720525	0.459	A	I	0.245	5.98E-05	ABCG1
rs10798331	1	175034437	0.311	A	O	-0.2462	6.01E-05	TNN
rs1278898	14	32401608	0.48	T	I	0.245	6.04E-05	
rs9705829	12	34827512	0.173	C	I	-0.245	6.04E-05	
rs1278905	14	32402699	0.487	T	I	0.245	6.10E-05	
rs1057640	14	103566904	0.228	A	O	0.2481	6.19E-05	EXOC3L4
rs1278907	14	32403863	0.484	A	I	0.245	6.27E-05	
rs2133308	3	159676705	0.248	A	I	-0.245	6.32E-05	
rs7218421	17	31917615	0.197	A	I	-0.245	6.32E-05	ASIC2
rs9395487	6	49355974	0.386	A	I	0.244	6.51E-05	
rs11502507	12	34840819	0.173	A	I	-0.244	6.72E-05	
rs11827298	11	24324000	0.294	A	I	-0.244	6.76E-05	
rs6853433	4	129303369	0.173	G	I	-0.244	6.78E-05	
rs10820829	9	93898807	0.287	C	I	-0.244	6.79E-05	LOC100128909

rs7645203	3	159686669	0.385	T	I	-0.244	6.79E-05	
rs6538724	12	96930565	0.343	C	I	0.243	6.85E-05	C12orf55
rs7645223	3	159686752	0.385	G	I	-0.243	6.88E-05	
rs7320083	13	75229158	0.33	G	I	-0.243	6.89E-05	
rs7977582	12	96935343	0.354	C	I	0.243	6.89E-05	C12orf55
rs679143	1	39204184	0.203	A	O	-0.2438	6.91E-05	
rs1278904	14	32402580	0.492	A	I	0.243	6.91E-05	
rs2058899	2	71580129	0.374	A	O	0.2441	6.95E-05	ZNF638
rs6425317	1	175051843	0.316	A	I	-0.243	6.96E-05	TNN
rs9807155	18	53578214	0.421	A	O	0.2455	6.98E-05	
rs9309137	2	47011834	0.316	G	O	-0.2443	7.08E-05	
rs7520373	1	8397803	0.388	A	O	0.2429	7.10E-05	SLC45A1
rs73331622	12	34828263	0.173	A	I	-0.243	7.28E-05	
rs74357723	9	93965245	0.128	C	I	0.242	7.33E-05	FP2234
rs35313632	17	16779081	0.127	G	I	-0.242	7.37E-05	
rs13091882	3	143818661	0.423	T	I	0.242	7.46E-05	
rs4334120	12	24704118	0.212	T	I	0.242	7.60E-05	SOX5
rs3994821	1	8397522	0.353	A	I	0.242	7.65E-05	SLC45A1
rs17749748	2	71605145	0.377	G	I	0.242	7.70E-05	ZNF638/ZNF638-IT1
rs7980921	12	96935879	0.358	G	I	0.242	7.70E-05	C12orf55
rs10115776	9	126850913	0.281	T	I	-0.242	7.73E-05	
rs2551463	18	53619906	0.438	C	I	0.242	7.76E-05	
rs7217007	17	46844697	0.356	G	O	0.2426	7.76E-05	TTLL6
rs12474072	2	71636036	0.379	G	I	0.242	7.78E-05	ZNF638
rs9573428	13	75228536	0.331	C	I	-0.242	7.84E-05	
rs835745	11	44887757	0.273	G	O	-0.2438	7.85E-05	TSPAN18
rs12432953	14	32404503	0.485	C	I	-0.241	7.86E-05	LOC100506110
rs1808245	17	12804397	0.182	T	I	0.241	7.86E-05	ARHGAP44
rs220780	6	165818538	0.351	C	I	0.241	7.87E-05	PDE10A
rs1990696	12	96930179	0.351	A	O	0.2414	7.88E-05	C12orf55
rs6538725	12	96930610	0.351	G	I	0.241	7.91E-05	C12orf55
rs4531814	18	45590277	0.428	G	I	0.241	7.95E-05	ZBTB7C
rs112026874	2	163512171	0.208	T	I	-0.241	8.05E-05	KCNH7
rs3135132	4	2354353	0.309	A	O	0.242	8.07E-05	ZFYVE28
rs10819162	9	129225538	0.228	T	O	0.2418	8.20E-05	FAM125B
rs2911663	8	30596384	0.397	A	I	-0.241	8.24E-05	UBXN8
rs2234721	21	43710096	0.27	A	O	-0.2422	8.24E-05	ABCG1
rs7959244	12	24699489	0.214	G	I	0.241	8.28E-05	SOX5
rs9668792	12	34852633	0.142	T	I	-0.241	8.28E-05	
rs12801367	11	24321827	0.296	A	O	-0.2416	8.29E-05	
rs9992683	4	77147300	0.192	T	I	0.241	8.32E-05	
rs10991899	9	94118921	0.129	G	O	0.2406	8.34E-05	AUH
rs11201150	10	86414323	0.428	C	I	-0.241	8.35E-05	
rs3994814	1	8397518	0.352	A	I	0.241	8.36E-05	SLC45A1
rs4575574	17	12792623	0.183	G	I	0.241	8.37E-05	ARHGAP44
rs12802997	11	130576224	0.107	A	I	0.241	8.41E-05	C11orf44
rs7276395	21	43721210	0.457	A	I	0.24	8.45E-05	ABCG1
rs34262165	5	25028914	0.351	G	I	-0.24	8.46E-05	
rs4770928	13	26730858	0.247	T	I	-0.24	8.48E-05	
rs7610160	3	159686345	0.384	G	O	-0.2408	8.49E-05	
rs78079139	9	93958551	0.127	A	I	0.24	8.49E-05	FP2234
c7.157489602.b37p0	7	157489602	0.498	A	I	0.24	8.53E-05	PTPRN2
rs59791189	9	129204537	0.228	A	I	0.24	8.56E-05	FAM125B
rs56249723	2	163508193	0.209	T	I	-0.24	8.58E-05	KCNH7
rs1461349	11	37200926	0.344	A	O	-0.2448	8.59E-05	
rs6538723	12	96930341	0.349	A	O	0.2401	8.63E-05	C12orf55
rs1420619	12	96925106	0.354	C	I	0.24	8.69E-05	C12orf55
rs1940103	11	129668762	0.227	G	I	-0.24	8.69E-05	
rs4828051	23	99994917	0.319	A	I	0.24	8.70E-05	SYTL4
rs55942334	2	163507833	0.208	T	I	-0.24	8.73E-05	KCNH7
rs67301489	19	7256392	0.328	G	I	0.24	8.73E-05	INSR
rs17271094	23	116919706	0.373	A	O	-0.2399	8.76E-05	
rs12038661	1	8398834	0.39	G	I	0.24	8.77E-05	SLC45A1
rs2196268	2	163505633	0.207	T	I	-0.24	8.77E-05	KCNH7
rs73250616	23	99993277	0.319	C	I	0.24	8.78E-05	SYTL4
rs729184	14	103576415	0.256	A	I	0.24	8.79E-05	EXOC3L4
rs11108558	12	96904058	0.353	A	I	0.24	8.81E-05	C12orf55
rs1395376	18	53583258	0.415	A	O	0.2402	8.85E-05	
rs1506632	18	53579294	0.415	G	O	0.2402	8.85E-05	
rs7320397	13	26730629	0.247	G	I	-0.24	8.86E-05	
rs2323726	11	129671438	0.227	T	I	-0.24	8.88E-05	
rs6502210	17	12807337	0.185	C	I	0.24	8.88E-05	ARHGAP44
rs13023539	2	171319658	0.325	T	I	0.24	8.89E-05	MYO3B
rs7150145	14	55508260	0.365	A	I	0.24	8.92E-05	SOCS4
rs601667	1	39205701	0.216	T	I	-0.24	8.96E-05	
rs4882547	12	83529560	0.291	A	I	0.24	9.00E-05	TMTC2
rs11652580	17	12783663	0.186	G	O	0.24	9.01E-05	ARHGAP44
rs1530478	3	143819553	0.421	T	O	0.2394	9.12E-05	
rs9514612	13	107695526	0.145	G	I	0.239	9.19E-05	
rs12296410	12	24707604	0.214	A	O	0.2397	9.20E-05	SOX5/LOC100129937
rs10507385	13	28996960	0.203	G	O	-0.2397	9.21E-05	FLT1
rs7338740	13	75231803	0.349	C	I	-0.239	9.26E-05	
rs73447422	18	38576698	0.138	C	I	-0.239	9.37E-05	

rs6960504	7	124530314	0.382	A	I	0.239	9.40E-05	POT1
rs75785779	9	989868	0.234	C	I	-0.239	9.40E-05	DMRT3
rs4852782	2	71636240	0.379	G	O	0.2389	9.42E-05	ZNF638
rs6714975	2	71633389	0.379	A	O	0.2389	9.42E-05	ZNF638
rs9928779	16	80343571	0.344	C	I	0.239	9.47E-05	
rs15661	21	43724353	0.448	G	O	0.2401	9.52E-05	TFF3/ABCG1
rs4970621	1	39202012	0.193	G	I	-0.239	9.56E-05	
rs79936603	9	93958597	0.126	C	I	0.239	9.59E-05	FP2234
rs10029710	4	77164337	0.257	T	I	0.239	9.61E-05	FAM47E
rs4727743	7	110332746	0.454	A	I	0.239	9.62E-05	IMMP2L
rs4710723	6	170744106	0.429	A	O	-0.2399	9.63E-05	
rs10860048	12	96903584	0.354	G	I	0.239	9.64E-05	C12orf55
rs35246666	7	157490324	0.478	G	I	-0.239	9.64E-05	PTPRN2
rs6745907	2	71609073	0.376	G	I	0.239	9.66E-05	ZNF638/ZNF638-IT1
rs35362051	12	96931832	0.333	C	I	0.238	9.69E-05	C12orf55
rs1878817	16	80348231	0.37	A	O	0.2416	9.71E-05	
rs1077146	22	18349379	0.44	T	I	-0.238	9.73E-05	MICAL3
rs60805089	14	55503348	0.364	T	I	0.238	9.73E-05	SOCS4/WDHD1
rs6061677	20	60198066	0.377	T	O	-0.2384	9.74E-05	CDH4
rs6645920	23	116912834	0.327	G	I	-0.238	9.75E-05	
rs4936087	11	129669586	0.227	C	I	-0.238	9.76E-05	
rs4937462	11	129669391	0.227	C	I	-0.238	9.76E-05	
rs1990693	12	96931407	0.333	A	I	0.238	9.77E-05	C12orf55
rs7962433	12	96935361	0.362	G	I	0.238	9.79E-05	C12orf55
rs10400524	12	29654807	0.238	G	O	-0.2387	9.87E-05	TMTC1/OVCH1
rs7314259	12	10406567	0.212	C	I	-0.238	9.89E-05	
rs4852256	2	71631345	0.38	T	I	0.238	9.90E-05	ZNF638
rs11560879	11	129677367	0.228	A	I	-0.238	9.91E-05	TMEM45B
rs2402763	7	124709732	0.366	A	I	0.238	9.91E-05	
rs11047490	12	24698452	0.215	C	I	0.238	1.00E-04	SOX5
rs34831860	12	96912515	0.353	C	I	0.238	1.00E-04	C12orf55
rs35481501	17	16811940	0.135	T	I	-0.238	1.00E-04	
rs6061682	20	60209375	0.379	T	I	-0.238	1.00E-04	CDH4

Supplementary Table S2. SNPs that were assoicated with HHT AUC values with p<10E-3
Genotype*. I: imputed SNP; O: genotyped SNP
